# High Frequency Components of Hemodynamic Shear Stress Profiles are a Major Determinant of Shear-Mediated Platelet Activation in Therapeutic Blood Recirculating Devices

**DOI:** 10.1038/s41598-017-05130-5

**Published:** 2017-07-10

**Authors:** Filippo Consolo, Jawaad Sheriff, Silvia Gorla, Nicolò Magri, Danny Bluestein, Federico Pappalardo, Marvin J. Slepian, Gianfranco B. Fiore, Alberto Redaelli

**Affiliations:** 1 Department of Electronics, Information and Bioengineering, Politecnico di Milano, Milan, Italy; 2 Anesthesia and Cardiothoracic Intensive Care, IRCCS San Raffaele Scientific Institute, Vita Salute University, Milan, Italy; 3 Department of Biomedical Engineering, Stony Brook University, New York, USA; 4 Department of Medicine and Biomedical Engineering, Sarver Heart Center, The University of Arizona, Arizona, USA

## Abstract

We systematically analyzed the relative contributions of frequency component elements of hemodynamic shear stress waveforms encountered in cardiovascular blood recirculating devices as to overall platelet activation over time. We demonstrated that high frequency oscillations are the major determinants for priming, triggering and yielding activated “prothrombotic behavior” for stimulated platelets, even if the imparted shear stress has low magnitude and brief exposure time. Conversely, the low frequency components of the stress signal, with limited oscillations over time, did not induce significant activation, despite being of high magnitude and/or exposure time. *In vitro* data were compared with numerical predictions computed according to a recently proposed numerical model of shear-mediated platelet activation. The numerical model effectively resolved the correlation between platelet activation and the various frequency components examined. However, numerical predictions exhibited a different activation trend compared to experimental results for different time points of a stress activation sequence. With this study we provide a more fundamental understanding for the mechanobiological responsiveness of circulating platelets to the hemodynamic environment of cardiovascular devices, and the importance of these environments in mediating life-threatening thromboembolic complications associated with shear-mediated platelet activation. Experimental data will guide further optimization of the thromboresistance of cardiovascular implantable therapeutic devices.

## Introduction

Hemodynamic shear stress interacts with and impacts platelets leading to a spectrum of responses ranging from altered biochemical signaling, to activation, to complete destruction with microparticle formation^1^. Exposure to fluid shear stress alone has been demonstrated to activate and aggregate platelets irreversibly, without the need for exogenous agonists^2^. Shear-mediated platelet activation (SMPA) shows a consistent dose and time response, with stoichiometric characteristics similar to those of chemical agonists^3–6^.

In particular, the supraphysiological levels of hemodynamic shear stress, i.e. “hypershear”, exerted by therapeutic mechanical cardiovascular blood recirculating devices (BRDs), such as ventricular assist devices (VADs), the total artificial heart, and prosthetic heart valves (PHVs), expose circulating platelets to severe mechanical loading regimens, enhancing SMPA^7–9^. Hypershear stress in BRDs is associated with altered flow conditions, such as adverse pressure gradients, flow separation, vortex formation, vortex shedding and turbulence, which also contribute to platelet activation^8^. Two further effects contribute to SMPA in BRDs: first, platelet damage increases with repetitive passes through a device, suggesting that platelets accumulate cyclic shear stress exposures; second, shear stress also has a sensitizing effect on platelets, so that platelets exposed to very high shear stress - even for brief exposures, such as during passage through BRDs - continue to activate despite subsequent exposure to low shear stress - as is encountered downstream of BRDs - with a residual incremental response compounding that of the initial high shear pulses or waveforms^10–12^.

Shear flow-related mechanisms of SMPA are likely to have predominant implications in the development of thromboembolic complications in patients implanted with mechanical therapeutic BRDs^13–15^, which contribute significantly to overall morbidity and mortality^16, 17^.

Due to the complex nature of hemodynamic shear stress patterns in BRDs, characterized by differing and highly time-varying shear stress magnitudes, pulse durations, directions and temporal oscillations, the identification of the dominant hemodynamic characteristics that drive altered platelet function is challenging. In last few years, different approaches and methodologies have been developed to elucidate the mechanisms driving SMPA and to identify possible design solutions to refine and minimize the device-associated thrombogenicity. These include numeric and experimental tools, often used in combination, to model shear stress waveforms and to predict and characterize the phenomenological platelet response^18–20^.

In the context of this research area, newer, and progressively more refined phenomenological models of SMPA have been advanced^21, 22^. More recently, we defined a new class of SMPA model of the differential-type^23^. This model was based on a general reaction equation, including terms accounting for different hemodynamic characteristics of the shear stress, namely the shear stress magnitude and the corresponding exposure time, which together define the stress accumulation (SA, i.e., the integral of the scalar shear stress over time), and the stress rate (SR, i.e. the shear stress loading rate, or the variation of the shear stress over time). This model successfully described experimental observations of platelet response to a variety of time-constant and dynamic shear stress conditions, also properly describing platelet sensitization^23^. Moreover, this model allowed prediction of the logistic saturation of platelet activation. In fact, as observed experimentally, at high numbers of repetitive stimulation cycles, the activation rate progressively vanishes and platelets reach a maximum activation level asymptotically^23^. However, the model formulation was defined through *in vitro* studies that examined the platelet activation response to synthetic shear stress loading waveforms that differed significantly from loading conditions relevant to devices, thus warranting further investigation aimed at validating its true strength.

In the present study, we evaluated the dynamics of SMPA *in vitro*, induced by realistic, i.e., BRD-like, hemodynamic shear stress patterns. We hypothesized that deconstructing and analyzing clinically accurate hemodynamic stress waveforms associated with platelet passage through BRDs would yield component elements, including: the distribution of frequency components, SA and SR, that would be more predictive of platelet activation than use of the waveform alone as a whole. As such, first we exposed platelets to shear flow mechanical loading characteristics of a recently developed trileaflet PHV and measured the dynamics of activation in response to repetitive shear stress exposure. We focused our analysis on characterizing the mutual contribution of SA and SR on SMPA. For this aim, we used principles of signal frequency content analysis. In fact, both SA and SR are hemodynamic characteristics that can fundamentally be described by the frequency spectrum of a given shear stress waveform. In detail, while low-frequency components of the spectrum are associated with a dominant contribution of SA (high magnitude and exposure time, low temporal oscillation), high-frequency components are associated with high SR (high temporal oscillation, low magnitude and exposure time). In view of that, we selected specific frequency components of the spectrum of two PHV-shear stress profiles (from low- to high-frequency components) to obtain a set of test-curves yielding different values of SA and SR, respectively.

We then tested the predictive capability of the differential-type numerical model^23^ of SMPA in response to the realistic PHV shear stress waveforms by comparing experimental data with numerical predictions of platelet activation. Moreover, we compared the experimental dynamics of platelet activation of the extracted frequency waveforms with numerical predictions, to test the ability of the numerical model to discriminate between the distinctive effects mediated by SA and SR and to properly balance their mutual contribution to SMPA.

## Methods

### Definition of the test-curves

**Figure 1:**
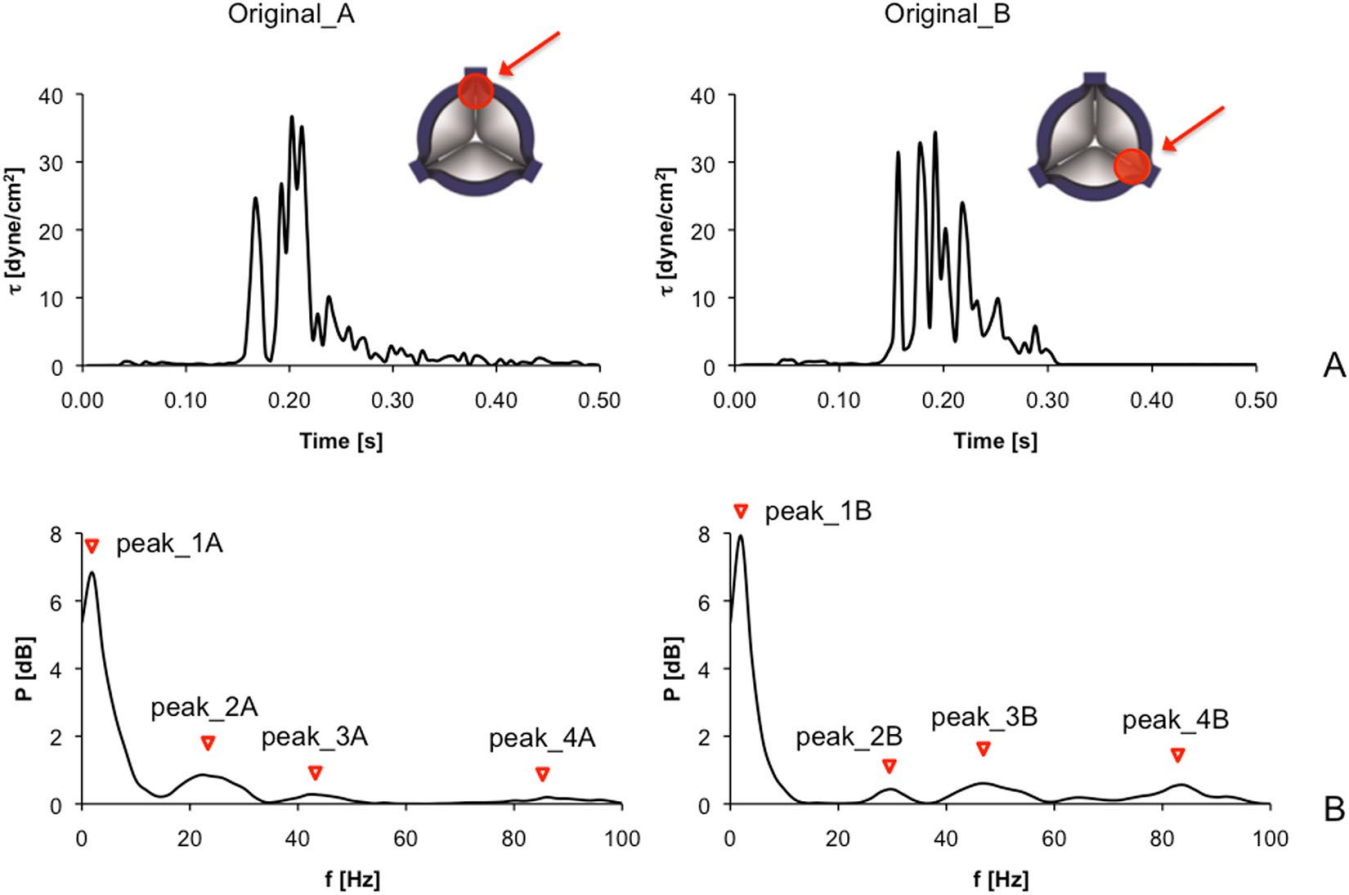
(**A**) Hemodynamic shear stress profiles characteristics of the trileaflet PHV are shown in the time-domain: the two curves, named Original_A (left) and Original_B (right) were computed from simulated trajectories of platelets flowing through two selected gap regions between the leaflet and the valve annulus^24^, indicated by arrows. (**B**) Power spectrum (PS) of the curves allows representing Original_A (left) and Original_B (right) in the frequency-domain: the principal frequency components of the PS are indicated by red triangles (Peak_1A–4A, Peak_1B–4B).

Two hemodynamic shear stress profiles characteristics of a recently developed trileaflet polymeric PHV^24^ were utilized to define a set of paradigmatic BRD-like test-curves for both the experimental as well as the numerical characterization of the platelet response. These profiles are named Original_A and Original_B, and are shown in Fig. 1A. Those curves were extracted by combining: i) a Fluid Structure Interaction (FSI) algorithm allowing to model the valve kinematics over consecutive cardiac cycles and ii) a Lagrangian formulation to track the trajectory of tens of thousands of injected platelets passing through specific regions of interest (ROI) of the valve^24^. Then, a Probability Density Function (PDF) of the corresponding shear stress curves was built (i.e., the PHV thrombogenic footprint) and the most frequently occurring (mode) SA value of the PDF was selected as the most representative shear stress pattern of each ROI^24^ (i.e., the Original_A and Original_B curves).

The frequency content of Original_A and Original_B was extracted through Fourier analysis, where a Fast Fourier Transform (FFT) algorithm was performed to convert the two curves from their time domain to a representation in the frequency domain^25^. Principal frequency components of the two shear stress waveforms were identified from the power spectrum (PS) curve (Fig. 1B). The PS describes the distribution of the principal frequency components of a given signal and the power associated with the signal at each frequency and is calculated as the square of the absolute values of the FFT coefficients. The PS was calculated with a frequency-step equal to the reciprocal of the time period (Δt) of the considered curves. The PS for Original_A and Original_B, which have a Δt equal to 0.5 s^24^, was thus computed with a 2-Hz frequency step. The PS was then discretized in frequency bands (FBs) using the “find peaks” tool of Matlab (The MathWorks, Inc., USA). Each FB was calculated around each of the identified principal frequency component of the PS, corresponding to the local maxima of the spectrum. In detail, each FB was calculated as the full width at half-maximum amplitude of each peak. According to our analysis, for each waveform, four principal components (Peaks) were identified (red triangles in Fig. 1B). Data on FB values and power signal (P) for each of the four peaks (Peaks 1–4) identified from the PS of Original_A and Original_B are reported in Table 1.

**Table 1:** Frequency bands (FB) and power signal (P) of the four peaks identified from the power spectrum of the Original_A and Original_B curves.

	FB [Hz]	P [dB]
Original_A		
Peak_1	0–6	6.83
Peak_2	18–30	0.86
Peak_3	38–50	0.28
Peak_4	82–94	0.20
Original_B		
Peak_1	0–6	7.92
Peak_2	28–36	0.43
Peak_3	40–56	0.60
Peak_4	82–94	0.56

**Figure 2:**
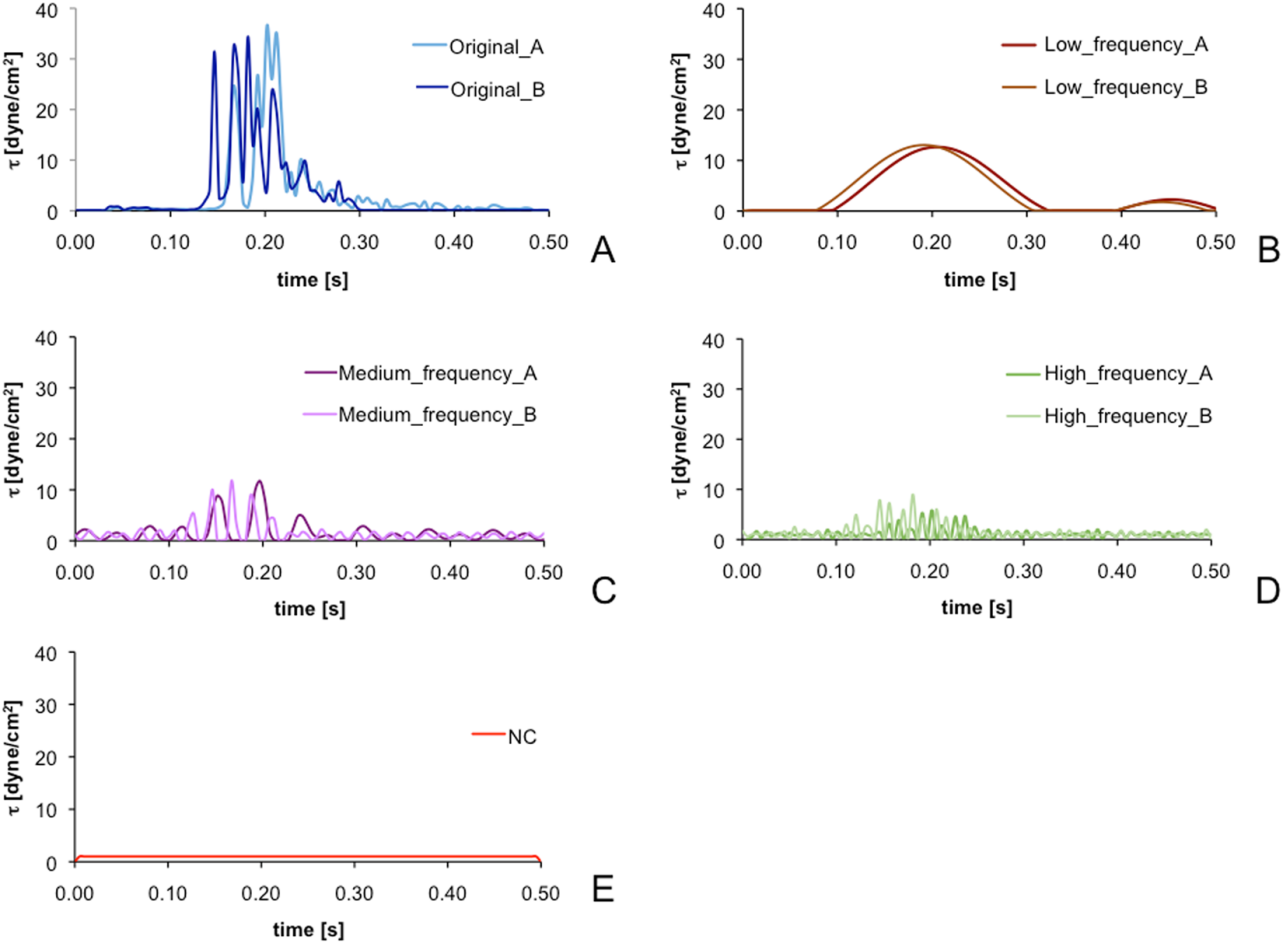
The test-curve data set: (**A**) Original curves; time-domain reconstructed curves corresponding to the (**B**) low- (Peak_1A and Peak_1B), (**C**) medium- (Peak_2A and Peak_3B) and (**D**) high-frequency (Peak_4A and Peak_4B) component elements of the power spectrum of Original_A and Original_B; (**E**) the time-constant and low-stress amplitude negative control (NC) curve.

For both waveforms (i.e., Original_A and Original_B) three different Peaks were selected to cover the full frequency range, namely: i) the low-frequency peak (Peak_1A and Peak1B), ii) the medium-frequency peak with higher power signal (Peak_2A and Peak_3B for Original_A and Original_B, respectively), and iii) the high-frequency peak (Peak_4A and Peak4B), and corresponding time-domain curves were reconstructed through inverse FFT (Fig. 2). In this way, starting from the Original shear stress profiles, we obtained a set of test-curves modulating SA and SR, respectively. Moreover, a time-constant (SR = 0) and low-stress amplitude (1 dyne/cm^2^) waveform was included in the data set and used as a negative control (NC); the temporal duration of the NC was set to 0.5 s to match that of the dynamic test waveforms.

In Table 2, we reported SA and SR values for each of the test-curves of our data set. As the considered curves discrete signals, their SA and SR values were calculated according to (eqs 1 and 2):

1 $${\rm{SA}}=\sum _{{\rm{1}}=1}^{{\rm{N}}}\frac{{{\rm{\tau }}}_{{\rm{i}}}+{{\rm{\tau }}}_{{\rm{i}}+1}}{2}{\rm{\Delta }}{\rm{t}}$$

2 $${\rm{SR}}=\frac{1}{{\rm{N}}}\sum _{{\rm{i}}=1}^{{\rm{N}}}\frac{|{{\rm{\tau }}}_{{\rm{i}}+1}-{{\rm{\tau }}}_{{\rm{i}}}|}{{\rm{\Delta }}{\rm{t}}}$$

SA (eq. 1) was calculated as the integral of the scalar shear stress over time; SR (eq. 2) was calculated as the average shear stress time-gradient, which was selected as that representative of the time-varying characteristics of each curve. To calculate SR, we considered both the positive and negative variations of shear stress over time. Accordingly, SR accounts for the mechanical loading exerted on platelets during both acceleration (SR > 0) and deceleration (SR < 0) phases of the shear stress.

**Table 2:** Values of Stress Accumulation (SA) and Shear Stress Loading Rate (Stress Rate, SR) calculated for each of the test curves of our data set are compared with respect to values characteristics of the Original curves. The low-frequency curves (Peak_1A and 1B) show higher SA than Original curves but lower SR; SA progressively decreases while SR further increases in medium- (Peak_2A and 3B) and high-frequency curves (Peak_4A and 4B). The negative control (NC) curve has the lowest SA and null SR.

	SA [dyne · s/cm^2^]	SR [dyne/(cm^2^ · s)]
Original_A	1.64	425
Peak_1A	1.84	59
Peak_2A	0.78	173
Peak_4A	0.59	244
Original_B	1.64	518
Peak_1B	1.85	59
Peak_3B	0.71	275
Peak_4B	0.69	349
NC	0.50	0

**Figure 3:**
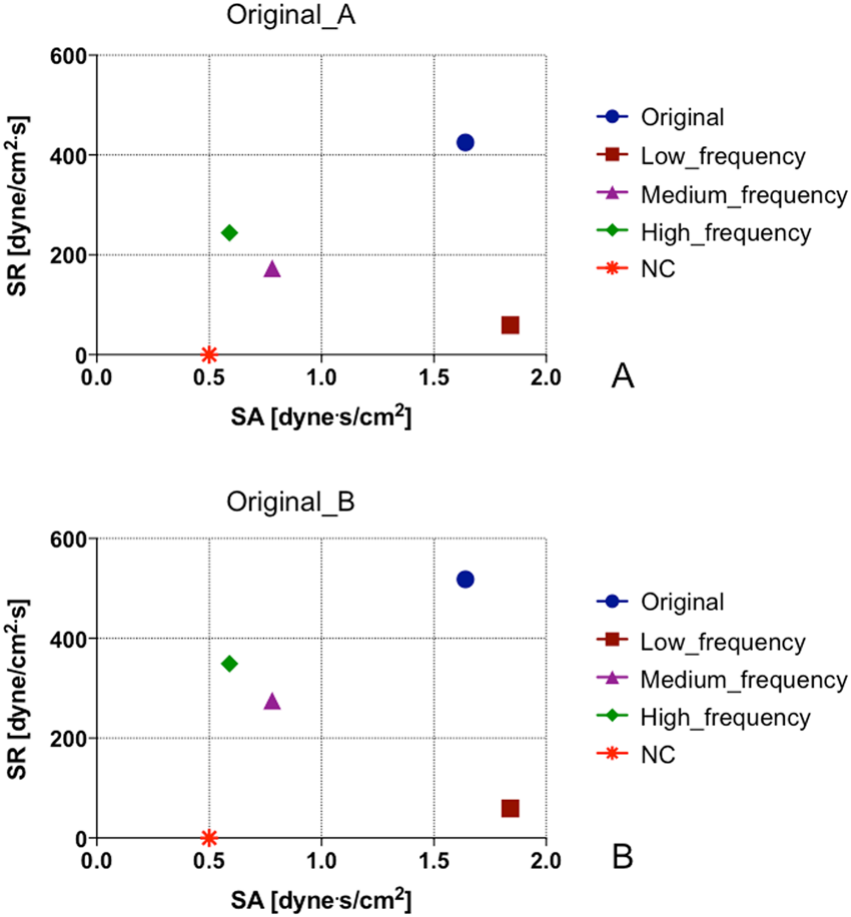
Values of SA and SR of each of the test-curves: by selecting specific frequency components of the spectrum of Original curves, we obtained a set of shear stress waveforms modulating the SA and SR values. (**A**): Original_A; (**B**) Original_B.

As shown in the SR *vs* SA chart of Fig. 3, the Original waveforms, being composed of all the frequency peaks of the PS, are characterized by high values of both SA and SR; the low-frequency waveforms have the highest value of SA (even greater than that of the Original) but the lowest value of SR (except for the NC curve); SA decreases and the SR increases progressively with increasing frequencies, i.e., from medium-to high-frequency waveforms. The high-frequency waveforms have an extremely low value of SA, comparable to that of the NC curve, but differ significantly from NC in terms of SR values. As such, while SA is a major hemodynamic characteristic of low-frequency waveforms, SR becomes progressively dominant for the medium- and high-frequency curves.

### Experimental characterization of the dynamics of shear-induced platelet activation

Whole blood (250 mL) was drawn through venipuncture from living adult bovine, collected into a blood bag (TERUFLEX, Terumo Corporation, Tokyo, Japan) containing 35 mL of citrate phosphate dextrose adenine (CPDA-1) as anticoagulant and transferred to the laboratory. Blood sampling procedures were performed in accordance with the European Commission directive (2010/63/UE) and with the laws and regulations on animal welfare enclosed in D.L.G.S. 26/14 and approved by the Italian Health Department. Purified gel-filtered platelets (GFP) were prepared from bovine whole blood and diluted to a standard concentration of 20,000 platelets/μl in a Hepes-modified Ca^2+^-free Tyrode’s buffer containing 0.1% fatty-acid-free bovine serum albumin, with 3 mM Ca^2+^ added 10 min prior to experiments^26^. Equilibrated GFP were maintained with gentle agitation at room temperature and used for stimulation experiments within 4 hours of gel-filtration.

The time-domain shear stress waveforms of the data set - specifically Original_A, Peak_1A, Peak_2A, Peak_4A; Original_B, Peak_1B, Peak_3B, Peak_4B, and the NC (Table 2) - were programmed into a computer-controlled Hemodynamic Shearing Device (HSD) that was used to stimulate GFP^19, 21^. Each curve was replicated consecutively to obtain a total of 10-min of stress exposure, corresponding to 600 consecutive repetitions of the shear stress profile.

For each stimulation test, 3 ml GFP were gently added to the HSD stimulation chamber and subsequently exposed to programmed shear stress, as outlined above. At pre-determined time-points, i.e. 2, 5 and 10 min - corresponding to 120, 300 and 600 repetitions - a platelet sample (50 μl) was withdrawn from the HSD and assayed via the modified prothrombinase Platelet Activity State (PAS) assay^11, 12, 27–30^, to quantify the associated level of activation (for details see Supplementary Information). In addition, a platelet sample was assayed immediately (at 0 min) after placing GFP within the HSD chamber, though prior to and without shear exposure, so to define baseline activation values of non-stimulated platelet samples.

GFP sampling from the HSD was performed automatically through a syringe pump (PSD/8, Hamilton, Reno, NV, USA). Briefly, a 250-μl glass syringe with a Luer fitting was mounted on the pump head and connected to the HSD stimulation chamber via a polyethelene tubing (2-mm inner diameter). The pump commands were programmed in LabView 2010 (National Instruments, Austin, TX, USA) and transmitted via an RS-232 port connected to the pump. When the pump was operated, 50 μl of GFP were drawn from the HSD stimulation chamber and collected into polyethelene conical tubes; slow sampling and collection flow rates (10 μl/s) were set to avoid any undesired GFP activation induced by the sampling and collection steps.

For each time-point, multiple PAS readings were performed to evaluate data consistency (*n* = 4). Furthermore, for each curve, stimulation tests were replicated with *N* = 3 different blood donors, on different experimental days, with each curve replicated twice on the same experimental day.

PAS values were plotted against time-of-stimulation to evaluate differences in the dynamics of activation induced by the different test-curves over time.

### Numerical characterization of the dynamics of shear-induced platelet activation

Experimental values of platelet activation were compared with those estimated using the three-term differential numerical PAS model^23^. Briefly, the model is structured to account for experimental observations of platelet activation rate tapering due to refractoriness, such that PAS saturates at a value of 1 as (eq. 3):

3 $$\frac{{\rm{dPAS}}}{{\rm{dt}}}={{\rm{K}}}_{0}[{\rm{PAS}},{{\rm{\tau }}}^{({\rm{t}})}({\rm{s}})](1-{\rm{PAS}})$$

where K_0_ is interpreted as the rate of stress-induced PA when PAS = 0 and ${{\rm{\tau }}}^{({\rm{t}})}({\rm{s}})={\rm{\tau }}({\rm{t}}-{\rm{s}}),{\rm{s}}\in [0,{\rm{t}}]$ as the scalar stress history of the platelet up to time t.

The shear-dependent platelet activation term of eq. 3 can be further expanded into three different and distinct terms (eq. 4):

4 $${{\rm{K}}}_{0}[{\rm{PAS}},{{\rm{\tau }}}^{({\rm{t}})}({\rm{s}})]={\rm{S}}({\rm{PAS}},{{\rm{H}}}_{{\rm{\tau }}})+{\rm{F}}({\rm{PAS}},{\rm{\tau }})+{\rm{G}}({\rm{PAS}},\dot{{\rm{\tau }}})$$

The term S(PAS, H_τ_) accounts for platelet sensitization, which is dependent on the current level of activation and on the stress accumulation of the entire stress history, H_τ_, and is defined as (eq. 5):

5 $${\rm{S}}({\rm{PAS}},{{\rm{H}}}_{{\rm{\tau }}})={{\rm{S}}}_{{\rm{r}}}{\rm{PAS}}\cdot {{\rm{H}}}_{{\rm{\tau }}}$$

where Sr is a constant that characterizes the sensitization response. The product of the current level of platelet activation and stress accumulation, PAS · H_τ_, linearly correlates with sensitization, as experimentally.

The entire stress history until time t (H_τ_) is defined as (eq. 6):

6 $${{\rm{H}}}_{{\rm{\tau }}}={\int }_{0}^{{\rm{t}}}{\rm{\tau }}({\rm{s}}){\rm{ds}}$$

The second and third terms of eq. 4, F(PAS, τ) and G(PAS, $\dot{{\rm{\tau }}}$), provide nonlinear rates of activation which are dependent on the current levels of stress and stress rate, respectively. These contributions to the activation rate are nonlinear due to their explicit dependence on PAS. The specific form of the former term utilizes the well-established power law behavior to describe low levels of PA under constant shear stress conditions, while the latter term provides a properly describes the phenomenological shear rate effect on PA as experimentally determined^23^. These terms are defined as (eqs 7 and 8):

7 $${\rm{F}}({\rm{PAS}},{\rm{\tau }})={{\rm{C}}}^{\frac{1}{{\rm{\beta }}}}{{\rm{\beta }}\mathrm{PAS}}^{\frac{{\rm{\beta }}-1}{{\rm{\beta }}}}{{\rm{\tau }}}^{\frac{{\rm{\alpha }}}{{\rm{\beta }}}}$$

8 $${\rm{G}}({\rm{PAS}},\dot{{\rm{\tau }}})={{\rm{C}}}_{{\rm{r}}}^{\frac{1}{{\rm{\delta }}}}{{\rm{\delta }}\mathrm{PAS}}^{\frac{{\rm{\delta }}-1}{{\rm{\delta }}}}{|\dot{{\rm{\tau }}}|}^{\frac{{\rm{\gamma }}}{{\rm{\delta }}}}$$

where C, α, β, Cr, δ, and γ are constants.

Numerical PAS values were computed according to constant values reported previously^23^ (Table 3). In addition, we systematically analyzed the contribution of each of those parameters and defined a new set of values (Table 3) in order improve fitting of the experimental data (see Results).

**Table 3:** Values of the model parameters used for the numerical prediction of shear-induced platelet activation dynamics.

	Sr	C	α	β	Cr	γ	δ
Original^23^	1.5701 × 10^−7^	1.4854 × 10^−7^	1.4854	1.4401	1.3889 × 10^−4^	0.5720	0.5125
Optimized	1.5701 × 10^−7^	1.4854 × 10^−7^	0.71825	0.72005	2.78 × 10^−5^	0.75	0.5125

Numerical PAS values were computed for all the considered stress histories, i.e. the two Originals, the low-, medium- and high-frequency curves, and the NC. For each curve, PAS was calculated at 0, 2, 5 and 10-min simulated time-points, allowing comparison of numerical predictions with experimental data.

### Statistical Analysis

Experimental PAS data are presented as mean ± standard deviation (SD). The Shapiro-Wilk normality test and the F-test of equality of variances were utilized to evaluate the data distribution; accordingly, results were analyzed through: i) parametric One-Way ANOVA on Ranks with Tukey’s *post*-hoc test, or the non-parametric Kruskal-Wallis One-Way ANOVA on Ranks with Dunn’s *post*-hoc test, to evaluate the dynamics of PAS over time associated with each of the tested curves, and ii) parametric Student’s *t*-test or the non-parametric Mann-Whitney *U* test to compare the PA level induced by different test-curves. Analyses were performed with Prism 7.0 (GraphPad Software, Inc., La Jolla, CA, USA). A *p* value < 0.05 was accepted as statistically significant.

## Results

Experimental platelet activation values of HSD-stimulated platelets, as measured through the PAS assay, and numerical predictions of SMPA, computed according to the differential-type numerical model^23^, are listed in Table 4: data are reported for each of the defined measurement time-points (0, 2, 5 and 10 min).

**Table 4:** Experimental and numerical values of PAS: data are reported for the whole data set of test curves for each of the defined measurement time points: t_0_: 0 min, t_2_: 2 min, t_5_: 5 min, t_10_: 10 min; exp: experimental values: num: numerical predictions according to^23^; num_opt: numerical predictions obtained with the optimized set of model parameters defined in this study.

	PAS [%]									
		NC	Original_A	Peak_1A	Peak_2A	Peak_4A	Original_B	Peak_1B	Peak_3B	Peak_4B
t_0_	exp	0.16 ± 0.14	0.30 ± 0.31	0.03 ± 0.04	0.88 ± 0.52	0.40 ± 0.23	1.45 ± 0.70	0.30 ± 0.11	0.79 ± 0.30	0.29 ± 0.17
	num	0.00	0.00	0.00	0.00	0.00	0.00	0.00	0.00	0.00
	num_opt	0.00	0.00	0.00	0.00	0.00	0.00	0.00	0.00	0.00
t_2_	exp	0.13 ± 0.17	0.62 ± 0.14	0.39 ± 0.22	0.73 ± 0.51	0.36 ± 0.22	3.65 ± 2.60	0.78 ± 0.12	0.78 ± 0.21	3.09 ± 1.72
	num	0.04	6.22	1.91	3.65	4.07	7.18	1.91	4.96	5.14
	num_opt	0.02	4.72	0.79	2.09	2.44	4.80	0.79	3.23	3.34
t_5_	exp	0.49 ± 0.17	2.39 ± 0.55	0.54 ± 0.53	1.10 ± 0.39	0.62 ± 0.35	6.52 ± 3.02	1.17 ± 0.70	2.55 ± 1.74	5.69 ± 1.96
	num	0.09	10.26	3.41	5.90	6.52	11.51	3.39	7.98	8.18
	num_opt	0.02	7.20	1.28	3.31	3.79	7.09	1.27	5.06	5.28
t_10_	exp	0.74 ± 0.32	14.77 ± 1.96	0.79 ± 0.45	2.66 ± 0.75	2.49 ± 0.36	19.62 ± 3.28	0.61 ± 0.26	8.63 ± 3.37	9.52 ± 2.89
	num	0.20	15.53	5.68	8.76	9.42	16.66	5.66	11.61	11.88
	num_opt	0.02	10.21	1.91	4.67	5.39	8.98	1.89	7.15	7.45

### Experimental dynamics of platelet activation

**Figure 4:**
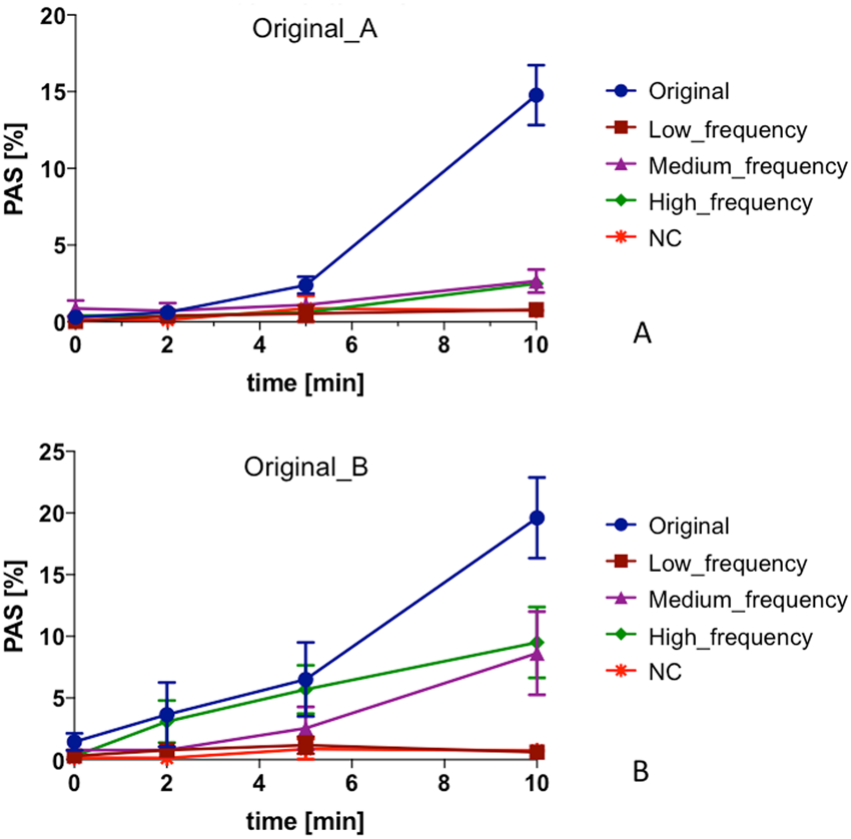
Experimental dynamics of SMPA (PAS values over time) of HSD-stimulated platelets as measured through the PAS assay; (**A**) Original_A; (**B**) Original_B.

According to our in *vitro* measurements of platelet activation (Fig. 4):

(i) The activation rate induced by the negative control (NC), which has null SR and low SA, was extremely low. Maximum PAS value at 10 min (PASt_10_) was equal to 0.74 ± 0.32% (<1%), thus indicating quite-negligible SMPA.
(ii) The Original curves, yielding high values of both SA and SR, induced a progressively increasing rate of activation with increasing stimulation time (*p* < 0.0001). The maximum experimental PAS value reached at 10 min (PASt_10_) was equal to 14.77 ± 1.96% and 19.62 ± 3.28% for Original_A and Original_B, respectively, significantly higher than PASt_10_ of the NC (*p* < 0.001). The two curves exhibited a non-linear trend of activation, where the first increase of the activation rate in the 0-to-5 min stimulation phase is followed by a further shift of the curve slope (5-to-10 min).
(iii) The low-frequency curves, with the highest SA but lowest SR, induced a small degree of SMPA over the considered stimulation period. The increase of PAS over time resulted in extremely low experimental values of PASt_10_ (0.79 ± 0.45% and 0.61 ± 0.26% for low_frequency_A and low_frequency_B, respectively), which were not statistically different from PASt_10_ of the NC (*p* > 0.05).
(iv) With an increase of SR, even if accompanied by a decrease of SA, as for medium- and high-frequency curves, experimental platelet activation revealed a consistent time-response: PAS over time was statistically different (*p* < 0.001) and maximum PASt_10_ was significantly higher compared to the NC (*p* < 0.001). A non-linear trend of activation was observed, with a shift in the activation rate after 5 min of stimulation.

### Numerical predictions of the dynamics of platelet activation

**Figure 5:**
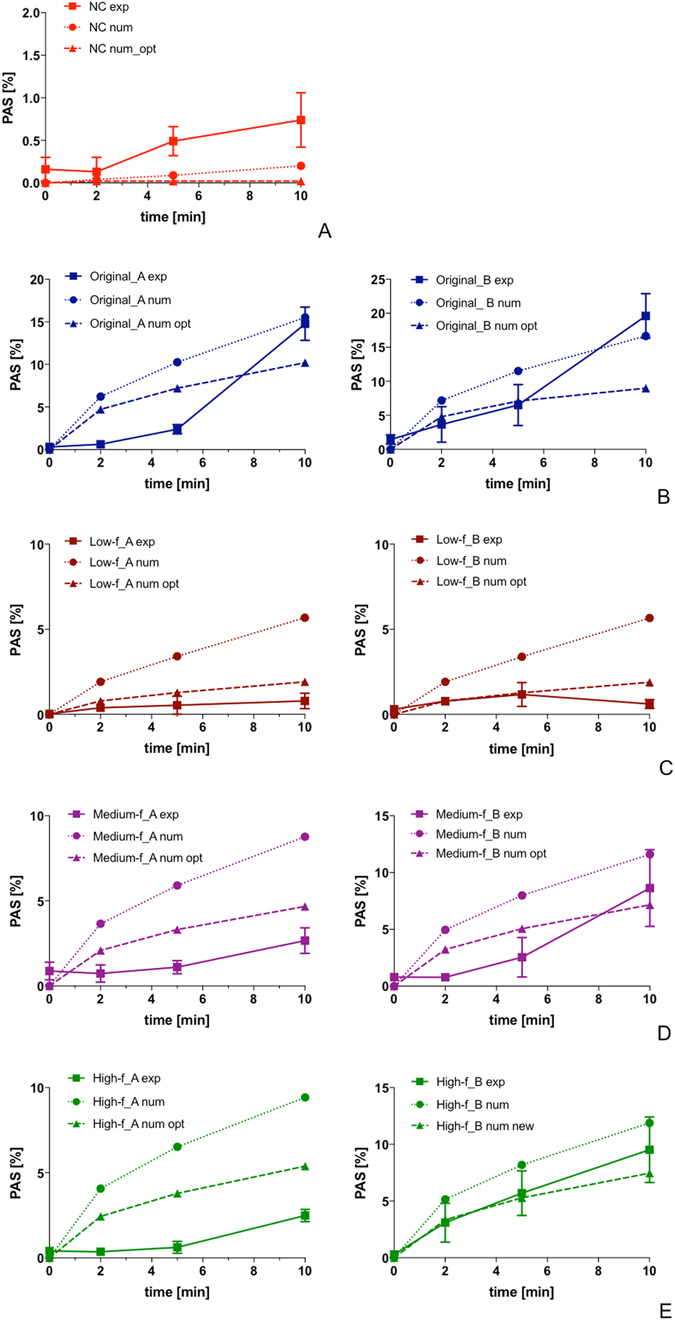
Comparison of numerical predictions of the dynamics of PA over time and experimental *in vitro* values: (**A**) NC curve; (**B**) Original curves; (**C**) low-frequency curves; (**D**) medium- frequency curves; (**E**) high-frequency curves.

According to our results obtained with previously-defined model parameters^23^ (Fig. 5):

(i) Numerical simulations predicted a non-incremental PAS rate induced by the NC after 10 min, thus suitably emulating the non-activating behavior observed experimentally (Fig. 5A);
(ii) The numerical activation trend of Original_A and Original_B showed a differing trend with respect to the experimental PAS dynamics (Fig. 5B). Numerical PAS values increased suddenly during the first 2 min of stimulation, followed by a decrease of the activation rate, whereas this was not seen *in vitro*. Experimentally, platelet activation remained fairly flat early, then rising rapidly after 5 min. Conversely, the numerical model neglected the rapid rise of the platelet activation rate that was observed *in vitro* after 5 min of stimulation. Despite these discrepancies, numerical values of maximum PASt_10_ were comparable to experimental results.
(iii) The numerical model predicted a non-negligible rate of activation over time for the low-frequency curves (Fig. 5C). The model overestimated maximum PAS (PASt_10_) with respect to experimental values: numerical PASt_10_ of low-frequency_A and low-frequency_B were 7.2-fold and 9.3-fold higher than experimental values, respectively.
(iv) The numerical model predicted a different activation trend for the medium- (Fig. 5D) and high-frequency (Fig. 5E) curves versus that which was observed experimentally. The model always predicted a sudden increase of platelet activation during the first 2 min of stimulation, which was not observed experimentally. Further, the model overestimated experimental maximum PASt_10_. Specifically, numerical PASt_10_ of medium-frequency_A and medium-frequency_B were 3.3-fold and 1.3-fold higher than *in vitro* values, respectively; numerical PASt_10_ of high-frequency_A and high-frequency_B were 3.8-fold and 1.2-fold higher than *in vitro* values, respectively.

The analysis of the contribution of each the model parameters in Equations 5–8 allowed to define a new set of constant values reported in Table 3 (labeled as “Optimized”). According to our results, although differences in the numerical activation trend persisted (i.e., sudden increase during the first 2 min of stimulation, followed by a decrease of the activation rate) previous large errors of numerical PAS values significantly decreased for nearly most of the frequency component elements of both Original A and Original B curves (Table 4, Fig. 5C–E). In detail, numerical PASt_10_ were: i) 2.54-fold and 3.09-fold higher for low-frequency A and B, respectively; ii) 1.75-fold higher and 0.82-fold lower for medium-frequency A and B, respectively; iii) 2.16-fold higher and 0.78-fold lower for high-frequency A and B, respectively. These values corresponded to a: i) 66% and 67% decrease for low-frequency A and B, respectively; ii) 47% and 38% decrease for medium-frequency A and B, respectively; iii) 42% and 37% decrease for high-frequency A and B, respectively, with respect to the results obtained with original model parameters^23^. On the other hand, while a good agreement between numerical and experimental PAS_10_ was previously observed for Original curves, the new set of constants leads to underestimation of the experimental values of both Original A and Original B (0.69-fold lower vs 1.05-fold higher and 0.45-fold lower vs 0.85-fold lower, with the optimized and original^23^ values, respectively).

### Analysis of the activating potential of SA and SR

**Figure 6:**
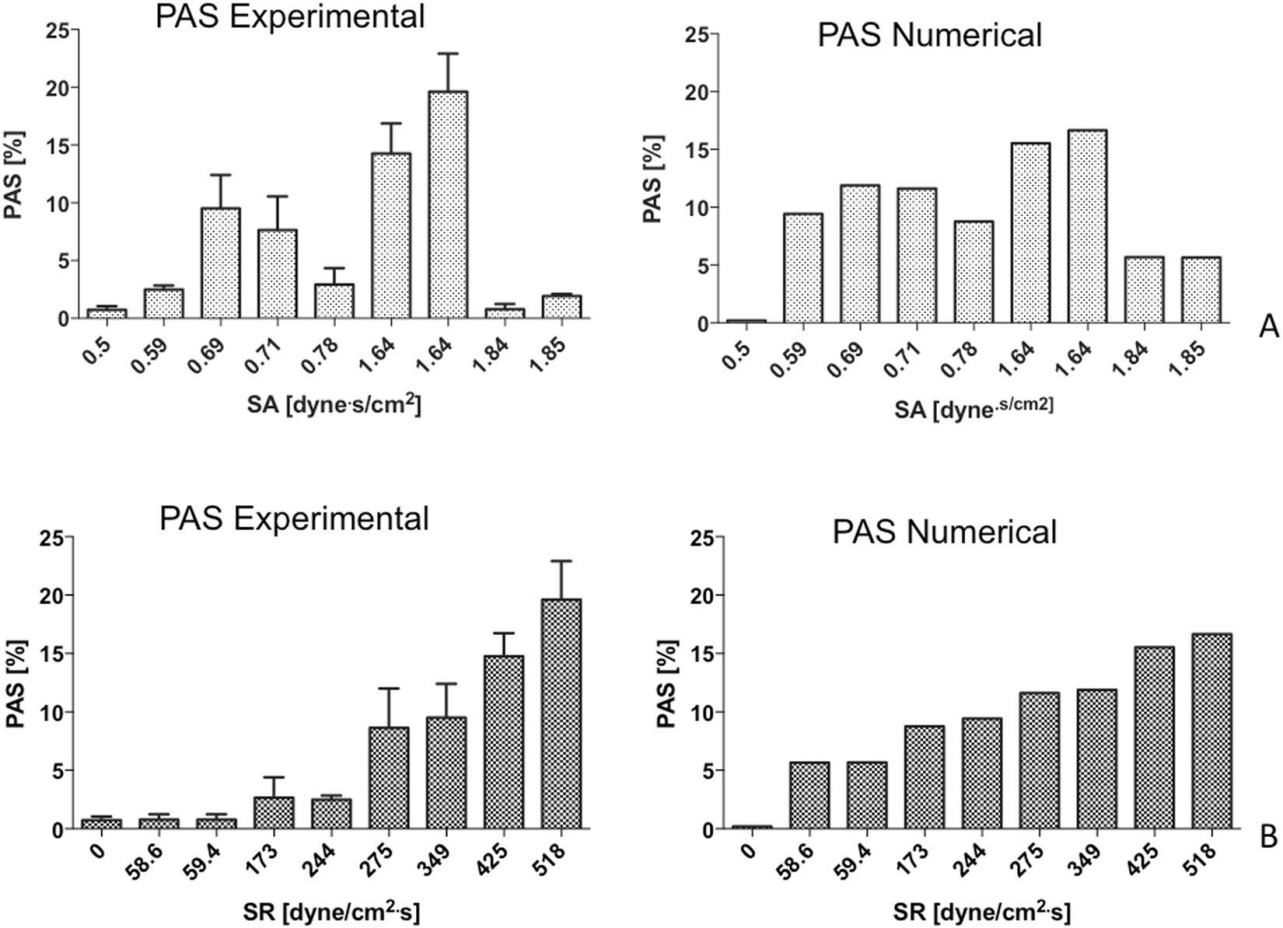
PAS values (mean ± SD) obtained after a 10-min stimulation period are plotted against values of SA (**A**) and SR (**B**) characterizing the curves of our data set; SA and SR are reported as for 1 stimulation cycle. Numerical PAS values are reported as computed according to ref. 23.

In Fig. 6, experimental and numerical PASt_10_ are plotted against values of SA (Fig. 6A) and SR (Fig. 6B) characterizing the shear stress profiles of our data set, allowing the selective evaluation of the effects of the two parameters on platelet activation. According to our experimental results, an increase in SA does not imply an increase of PAS (Fig. 6A). In contrast, our *in vitro* data demonstrated that PAS continues to increase with increasing values of SR (Fig. 6B). Consistent with this were the numeric, i.e. model results - specifically that a progressive increase in platelet activation was observed with increasing SR and that a more random association was observed for SA *vs* PAS, without a specific correlation detected.

**Figure 7:**
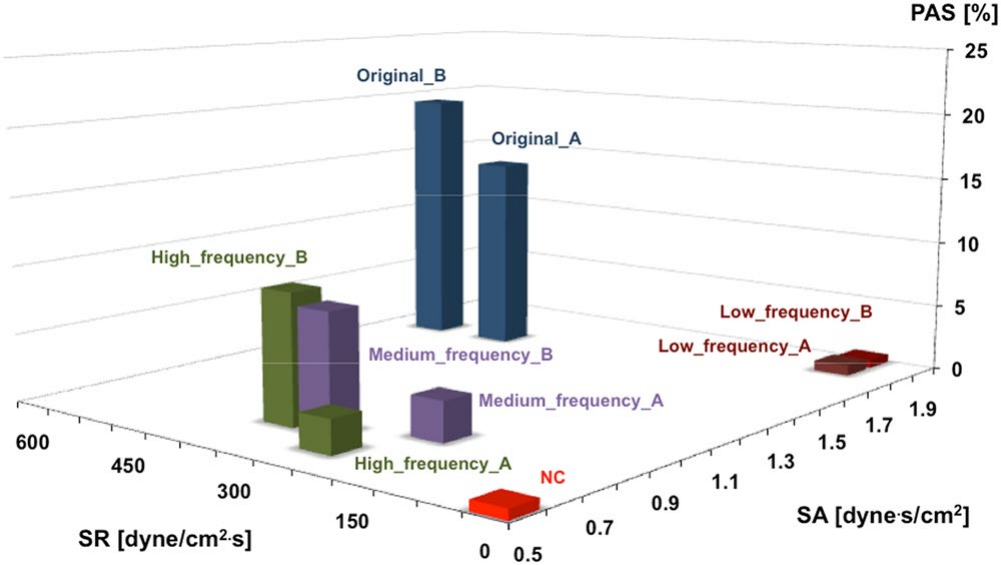
Experimental values of PASt_10_ are plotted in the SR-SA plane, allowing to comprehensively evaluate the effect of simultaneous variations of SA and SR in terms of induced PA: higher PAS values correspond to curves characterized by high values of both SA and SR; high values of SA alone do not induce any significant increase of PAS; increasing values of SR allows to significantly activate platelets. SA and SR are reported as for 1 stimulation cycle.

In Fig. 7, experimental values of PAS t_10_ are plotted in the SR-SA plane, allowing to comprehensively evaluate the effect of simultaneous variation of SA and SR on platelet activation. The results indicate that highest PAS values correspond to shear stress profiles characterized by high values of both SA and SR; high SA alone does not induce a significant increase of PAS; in contrast it is increasing SR that leads to progressively increased activation levels.

## Discussion

Thromboembolic complications in cardiovascular BRD recipients are typically multi-factorial involving elements of Virchow’s Triad, i.e. endothelial dysfunction and damaged vascular surfaces, inflammatory blood and abnormal flow, compounded by a host of patient-specific factors and issues related to lack of efficacy of anti-platelet agents, altered metabolism or insufficient administration of anti-thrombotic pharmacotherapy^31–33^.

Nonetheless, a dominating factor for thrombogenicity in BRDs are the non-physiologic hemodynamic blood flow patterns within these devices, which may induce and potentiate abnormal shear flow-related mechanical loading of platelets, eliciting SMPA. Thus, understanding the details of these abnormal flows and the critical components of this stress loading will advance our knowledge on platelet mechanobiology and provide translational insight as to how to improve BRD design. In fact, understanding the mechanisms that drive alterations of platelet function in BRDs may enhance the development of refined design solutions, mitigating the shear flow-mediated platelet damage, and minimizing the device-associated thrombogenicity.

The focal nature of platelet activation and thrombosis has long been attributed to the local hemodynamic shear stresses, but the exact components of the shear stress signal that contributes to activation with resultant thrombus formation have been, to date, difficult to decouple. As a matter of fact, a clear identification of the dominant hemodynamic characteristics of shear stress patterns responsible for SMPA in BRDs has as yet not been fully accomplished.

The present study extends our understanding of the impact of abnormal shear stresses on platelet activation. This is the first study, to our knowledge, that has specifically analyzed the thrombogenic potential of differing hemodynamic characteristics of shear stress profiles relevant to therapeutic cardiovascular BRDs. We systematically evaluated the platelet response to repetitive mechanical loading associated with the principal frequency components of two hemodynamic shear stress waveforms of a PHV. By applying experimentally extracted frequency waveforms (from low to high-frequency components), as well as the original PHV stress curves (composed of the full range of frequency components) we demonstrated that it is the high-frequency components that are the major determinants for priming, triggering and yielding activated “prothrombotic behavior” for stimulated platelets. Our results suggest that platelets are more sensitive to temporal oscillation of the shear stress (i.e., to high SR) even if the imparted shear stress has a relatively lower magnitude and/or briefer exposure time (i.e. low SA). Conversely, we showed that SA alone is unlikely to induce a significant activation rate when the contribution of SR is limited.

Our experimental results also show that while oscillation frequency (SR), amplitude and exposure time (SA) all together contribute to the platelet activity state, they do so though to differing degrees, i.e. with different powers. As observed in Fig. 4, the PAS values for Original waveforms (high SA and SR) are significantly greater (*p* = 0.001) than those associated with the extracted frequency waveforms at 10 min (high SA or high SR). Furthermore, the PASt_10_ values for medium- and high-frequency components of the Original_B waveform are more prominent than for Original_A. Examination of the devolved waveforms (Fig. 2) shows that the oscillation frequency of medium_frequency_B is greater than that of medium_frequency_A (i.e., higher SR, comparable SA), whereas the amplitude of several peaks of high_frequency_B is generally greater than that of high_frequency_A (i.e., higher SA, comparable SR), with some peaks exceeding 10 dyne/cm^2^.

The shear stress frequency spectrum proved to be a good predictor of platelet activation; allowing fractionation and decoupling of contributory components of shear stress signals and the subsequent systematic analysis of their contributions to overall platelet activation. Analysis of the hemodynamic components based on the signal frequency domain provides insight and may be useful in part to understand the activating potential of specific device-associated waveforms. In fact, according to our *in vitro* experimental results, the sum of PASt_10_ for frequency extracted component waveforms falls short of PASt_10_ for the Original waveforms, with none of the devolved waveforms approaching the amplitude (30–40 dyne/cm^2^) of the Original waveforms. These results suggest that the physical impact of shear stress provided by individual spectral frequency components, which are sensed and transduced by the platelet, may be less when acting individually versus when acting in concert as a full shear stress ensemble, resulting in less damage, poration, signaling or stimulation of mechanotransductive mechanisms - all vital biophysical mechanisms involved in platelet activation^1^. These results additionally generates hypothesis of reduced triggering of synergistic biochemical events, e.g. ADP release, being attenuated with only frequency component stimulation *vs* full spectrum stimulation.

In support of this differential stress-biochemical coupling effect, Feaver *et al*. investigated the inflammatory response of vascular endothelial cells as a function of the frequency content of wall shear stress in the carotid bifurcation^34^. The results from this study are in good agreement with our findings. Indeed, Feaver and colleagues showed that the inflammatory phenotype directly depends on specific components of the frequency domain of the shear stress profile^34^.

The shear stress patterns (i.e., the Original curves) used in this study were extracted by Piatti *et al*.^24^ by using a Lagrangian model formulation for tracking the platelet trajectories and for computation of the corresponding stress patterns. In this study we did not perform a direct comparison with different Lagrangian models presented in literature, since most of the existing Lagrangian models are based on the power law formulation of Grigioni *et al*.^35^, which Sheriff *et al*.^22^ showed is limited in capturing the shear-mediated platelet activation behavior in response to time-varying shear stress profiles. In addition, in the study by Piatti *et al*.^24^, a Lagrangian model - and not an Eulerian formulation - was considered as the aim of that study was to characterize the valve thrombogenic potential, i.e., to account for the activation of individual platelets, which may have a pro-coagulant propensity downstream of the valve. Indeed, as also reported in Hansen *et al*.^36^, Lagrangian methods better capture the accumulated stress histories of individual platelets, thus being better suited for the analysis of platelet activation when hot-spot values of shear stress are considered significant. Conversely, the continuum model underlying an Eulerian approach would inherently introduce smoothing effects on the computation of the shear stress. Furthermore, a Lagangian model formulation allowed us to experimentally test the platelet response to shear stress patterns using available equipment - i.e. the HSD, which relies on programmed Lagrangian stress waveforms.

Asymmetries can be observed in the shear stress profiles originating from the two commissural ROIs of the PHV we analyzed, despite the symmetry in the valve geometry (Fig. 1A). However, this is consistent when considering that variations in the stress–time waveforms of selected particle trajectories in the commissural ROIs can be related to non-negligible asymmetries in kinematics of the aortic leaflets of the valve. Moreover, the use of fluid structure interaction (FSI) algorithms may contribute in generating differences in the stress-time waveforms. Indeed, when FSI is used to solve high Reynolds number flow problems in regions of complex geometry, small numerical perturbations are sufficient to generate flow asymmetry. This phenomenon is similar to that attainable with direct numerical simulations (DNS) and is physically consistent. Computational FSI results for the aortic valve simulations were reported to be very close to experimental data^24^. In the study by Piatti *et al*.^24^, visual inspection of the aortic leaflets in the ViVitro left heart simulator set-up confirmed: i) differences in both leaflets fluttering and local surface curvature during systole^24^, and ii) a clear capture of the clockwise twisting of the leaflets free-margin during diastolic closure^24^. Accordingly, the systolic velocity field exhibits a centered but not completely symmetrical flow profile during ejection^24^.

In this study we were not necessarily concerned with specific locations in the region of the prosthetic valve, since this work was not directly aimed at characterizing thrombogenic “hotspots” in the valve. Accordingly, the regions selected to extract the Original shear stress curves - i.e., the two commissural regions, represent paradigmatic shear stress curves that we used to test our hypothesis (different frequency component elements of stress patterns drive different platelet activation dynamics). On the other hand, the two Original curves selected in this study were known to yield shear stress profiles that are more dynamic and have larger stress accumulation than the core flow region where the leaflets meet, thus being particularly fitting to the aim of this study. As such, we acknowledge that the experimental results of this study, although providing mechanistic results of platelet function in response to realistic shear stress waveforms encountered in BRDs, thus overcoming limitations of previous works where synthetic shear stress curves were used^22^, do not extend to a more clinically-pertinent endpoint - i.e., the characterization or the minimization of the thrombogenic potential of the specific valve/location in the valve.

For the measurement of the platelet activation level over consecutive shear-stress stimulation cycles we used the PAS assay, which required the use of gel-filtered platelets (GFP), i.e., a platelet suspension deprived of red and white blood cells and of plasma coagulation factors. Notably, the use of GFP eliminated other potential sources of platelet activation that might influence the description of the platelet-specific activation dynamics. Indeed, while the presence of red and white blood cells would increase activation effects due to cell collisions, the presence of plasma coagulation factors, which themselves play a role in activation and aggregation, would obfuscate the direct shear effect on platelet activation. Furthermore, aggregation or clot formation would reduce the available platelet count and change the flow dynamics during the platelet-stimulation tests in the HSD, therefore adding additional variables for which the PAS assay, as well as the numerical model we used for comparison of the experimental results, would not be able to account. In addition, with GFP we prevented clot formation, which would *per se* amplify the platelet activation levels. With the use of GFP we excluded all those co-factors contributing *in vivo* to platelet activation and characterized selectively the platelet response to shear. Further, the PAS assay, offered several advantages with respect to other platelet function tests that use whole blood or plasma, as it uses acetylated prothrombin (Ac-FII) as the substrate to quantify the platelet-mediated thrombin production rate: upon exposure to activated platelet membranes, Ac-FII is converted to thrombin, which is measured as a marker of activation. This represents the distinctive and more innovative feature of PAS assay: the modified thrombin produced by activated platelets does not further activate platelets^27^: as such, thrombin generation is a direct indicator of the level of activation. The presence of normal thrombin would prevent direct correlation with platelet activation levels, since thrombin is a potent agonist of platelet activation and would therefore amplify PAS levels with no correlation with the effective shear-mediated effect. In addition, the PAS assay allowed measuring the pro-thrombotic tendency of activated platelets without inducing aggregation or clot formation: those mechanisms are precluded due to the removal of plasma coagulation factors from GFP preparation. Furthermore, the thrombin produced by Ac-FIIa is inactive on fibrinogen and thus cannot mediate the conversion of fibrinogen to fibrin.

Concerning the results of the PAS numerical model, our data indicated that the differential-type PAS predictive model could effectively resolve a non-linear activation trend induced by complex device-like shear stress patterns. However, some differences have been noted with respect to experimental data. A good agreement in PAS values was found between numerical and experimental results for Original B and high_frequency B, but large errors were calculated for all other waveforms (Fig. 5). For nearly all the cases, numerical predictions overestimated *in vitro* PAS values. Conversely, the optimized constants we defined allowed significantly improved fitting of the experimental data, reducing the large errors of numerical PAS values for nearly all the frequency component elements of both Original A and Original B (Fig. 5). However, the analysis performed did not provide a universal set of model parameters that optimizes numerical results. While the new model coefficients improved the prediction for the single frequency components (Fig. 5), they lead to underestimation of PAS values of the Original curves (PASt_10_, Fig. 5). This is consistent with our experimental findings as we experimentally demonstrated that different frequency components all contribute to the overall PAS but with different powers (i.e., the overall PAS is not a linear combination or summation of effects).

Furthermore, numerical results displayed a concave behavior over time, whereas the experimental results showed a convex response (Fig. 5) and these differences persisted with the optimized model parameters. This discrepancy in the activation trend may be explained by several factors.

First, the model, which serves as a basis for the numerical simulations of this study, was developed for shear-mediated activation of human platelets, which showed a concave activating behavior at higher shear stresses and frequencies^22, 23^. In contrast, our work here has been performed with bovine platelets. Moreover, a prior study showed a lag in bovine platelet activation under constant shear when compared to human platelets^37^. Second, the model prescribes that the rate of activation increases over time following a logistic saturation process^23^, which was rarely observed experimentally, leading to overestimation of the early (i.e., 0-to-5 min stimulation time) experimental PAS values, and to a progressive decrease of the SMPA rate resulting in different dynamics of activation. While it is reasonable to assume that the platelet activation rate would reach an asymptote, whereby all the platelet activation components would be exhausted and no further activation would occur, experimental observations suggest that platelets have not reached this stage after the 600 stimulation cycles imposed via the HSD.

A further limitation of the numerical model is that it does not consider critical thresholds on shear stress amplitude, exposure time and stress variation^38^. The model places nearly equal emphasis (power) on the different hemodynamic characteristics composing the shear stress signal, thus failing to discriminate between different - and mutual contributions of SA and SR, overestimating, in particular, the effect associated with high SA (low-frequency). However, the current model formulation was tuned according to the experimental evolution of PAS in response to shear stress waveforms with higher SA than is used in these simulations^23^.

The model implies a more continuous, somewhat linear response to early shear stress stimulation. However, in biologic reality, it likely takes significant additive damage to cause platelets to become leaky, to sense and to effectively transduce the stress (i.e., to activate). Also, even if damage occurs somewhat early, membranes frequently repair themselves and self-seal^39, 40^, until a threshold of leak is exceeded leading to agonist and electrolyte influx and activation. Similarly, the lack of a rapid, progressive early rise of platelet activation for the low frequency signals (Fig. 5C) again reemphasizes the importance of intensity of injury and “membrane threshold” for damage needed to create poration, opening of channels or effective mechanical transduction. This explanation and concept extends to the observations seen for medium- and high-frequency components as well (Fig. 5C). As such, the numeric model assumes continuous progression of early activation, with the same “intensity of activation” occurring over the time progression. In contrast, in biological systems, with regard to platelet membrane and cytoplasmic dynamics, a threshold response is clearly occurring, hence an initial lag is followed by a much larger subsequent response with continued activation. This corresponds and translates to the kinetics of actin assembly, pseudopod extension and shape change occurring after a threshold is achieved, then accelerate though with eventual plateauing of the response anticipated.

On the other hand, numerical PAS results reflect the trend observed *in vitro* for both SA and SR (Fig. 6), although the normalized numerical PAS values are overestimated compared to the experimental PAS. The trend observations for SR confirm the earlier findings that an increase in PAS follows an increase in loading rate frequency^22^. The relationship between PAS and SA is not apparent in our study, and do not reflect the observation that an increase in SA leads to an increase in PAS^22^. However, it is important to note that the strong correlation between PAS and SA was achieved previously for more controlled constant or triangular shear stress waveforms^22, 23^.

To the best of our knowledge, no other numerical predictive models of platelet activation have been reported to account for shear stress amplitude and exposure time, stress oscillation frequency and accumulation of platelet damage. A simplified version of this numerical model, neglecting the contribution of stress rate, has been previously utilized in an Eulerian framework to show that platelet activation in abdominal aortic aneurysms accounts for less than 1% of background activation, with good agreement between the Eulerian model and a Lagrangian particle tracking approach^36^. This simplified model was further incorporated into a macroscopic predictive device-induced thrombus deposition and growth model to account for mechanically-activated paltelets^41^.

According to the results of this study, we advocate the need to further update the model formulation to specifically address numerical predictions of SMPA associated with loading conditions relevant to BRDs. In addition, comparison of bovine and human platelet behavior under realistic BRD-like shear should be accomplished, to eventually incorporate observed differences into the numerical model. Furthermore, the model should be integrated with different powers associated with shear stress magnitude, exposure time and stress variation, in order to better describe how those parameters individually impact, accumulate and effect platelets. Optimization of the numerical model would allow for better *in silico* estimation of platelet activation in BRDs for improving overall thromboresistance. Numerical estimation of platelet activation may be employed during the device design stage to compare the effect of modifications of specific geometric features conceived to reduce or eliminate critical flow conditions that elicit platelet activation and ultimately the overall device thrombogenic potential.

## Conclusion

This study provides a more fundamental understanding of the responsiveness of circulating platelets to the constitutive components of complex shear stress profiles. Further it provides enhanced understanding of platelet responsiveness to the hemodynamic environment of BRDs, and the importance of these environments in enhancing potentially life-threatening thomboembolic complications associated with shear-mediated platelet activation. We demonstrated that specific hemodynamic characteristics of hydrodynamic shear stress patterns, namely the shear stress oscillation frequency, are major determinants of shear-mediated platelet activation. Moreover, we showed that shear stress oscillation frequency, amplitude and exposure time together contribute to activate platelets, but with differing powers. This study provides observations which are useful in further defining basic mechanisms of the platelet mechanobiological response to shear stress. Further, our findings are useful in guiding future technological improvements of BRDs. Our experimental results will also serve as the basis for further refinement of state-of-the-art numerical PAS predictive models characterizing the shear-mediated phenomenological platelet response. Numerical predictive models of PA may represent a valuable, rapid and cost-effective platform to support and expedite the rational design optimization of local flow dynamics in BRDs.

## Electronic supplementary material

Supplementary Information
