# Supplementary material for: High Frequency Components of Hemodynamic Shear Stress Profiles are a Major Determinant of Shear-Mediated Platelet Activation in Therapeutic Blood Recirculating Devices
Source: Sci Rep. 2017 Jul 10;7:4994. doi: 10.1038/s41598-017-05130-5 (PMC5503983; doi:10.1038/s41598-017-05130-5)
Supplement: Supplementary file 1 — Supplementary Information [file 41598_2017_5130_MOESM1_ESM.pdf]

**Title:** High Frequency Components of Hemodynamic Shear Stress Profiles Are A Major Determinant of Shear-Mediated Platelet Activation In Therapeutic Blood Recirculating Devices

**Authors:** Filippo Consolo[1,2*], Jawaad Sheriff[3], Silvia Gorla[1], Nicolò Magri[1], Danny Bluestein[3], Federico Pappalardo[2], Marvin J Slepian[3,4], Gianfranco B Fiore[1], Alberto Redaelli[1].

**Affiliations:**

[1] Department of Electronics, Information and Bioengineering, Politecnico di Milano, Italy.

[2] Anesthesia and Cardiothoracic Intensive Care, IRCCS San Raffaele Scientific Institute, Vita Salute University, Italy.

[3] Department of Biomedical Engineering, Stony Brook University, USA.

[4] Department of Medicine and Biomedical Engineering. Sarver Heart Center. The University of Arizona, USA.

## SUPPLEMENTARY INFORMATION

### The Platelet Activity State (PAS) assay

The extent of activation of HSD-stimulated platelets was quantified via the PAS assay[27]. The PAS assay utilizes as substrate acetylated prothrombin, which upon exposure to damaged activated platelet membranes, as a result of elevated shear exposure, is converted to thrombin that is measured as a surrogate marker of activation[26]. The PAS assay allows characterizing the dynamic response of platelets by measuring the level of activation at increasing number of stimulation cycles[11,12,26-30].

To characterize the dynamics of activation, at each time step (0, 2, 5, and 10 min), 25 µl of GFP drawn from the HSD stimulation chamber were added to a 100-µl tube containing (final concentrations) 5,000 platelets/µl, 200 nM Ac-FII, 5 mM $Ca^{2+}$, and 100 pM FXa and the tube was incubated at 37°C for 10 min; then, a 10-µl sample was assayed for thrombin generation in a 96-wells microplate reader (Multiskan GO, Thermo Fisher Scientific Inc., Waltham, MA, USA), using 0.3 mM Chromozym-TH (Tosyl-Gly-Pro-Arg-4-nitranilide acetate, Roche Life Science, Milan, Italy) as the thrombin-specific chromogenic peptide substrate. Kinetic absorbance readings were performed at room temperature at 405-nm wavelength for 8 min[29]. The PAS value was calculated as the slope of the linear fitting of the absorbance-time data points over the 8-min kinetic reading. PAS values were normalized against those obtained by sonicating non-stimulated platelets with a microprobe sonicator (HD 2070 Sonoplus, Bandelin Electronic GmbH & Co. KG, Berlin, Germany). The sonication step is meant to yield platelets with maximal prothrombinase activity; thus, normalized PAS values (expressed in percentage, PAS [%]) represent the bulk activity as a fraction of the thrombin generation rate of sonicated platelets (100%)[28]. Sonication conditions (10 W for 10 s) were optimized for bovine platelets starting from the protocol reported for human platelets[28].
